# Does health technology assessment compromise access to pharmaceuticals?

**DOI:** 10.1007/s10198-022-01484-4

**Published:** 2022-06-16

**Authors:** Melanie Büssgen, Tom Stargardt

**Affiliations:** Hamburg Center for Health Economics, University of Hamburg, Hamburg, Germany

**Keywords:** AMNOG, Launch delay, Pharmaceuticals, HTA, Health policy, Price regulation, I18

## Abstract

In response to rapidly rising pharmaceutical costs, many countries have introduced health technology assessment (HTA) as a ‘fourth hurdle’. We evaluated the causal effect of HTA based regulation on access to pharmaceuticals by using the introduction of Germany’s HTA system (AMNOG) in 2011. We obtained launch data on pharmaceuticals for 30 European countries from the IQVIA (formerly IMS) database. Using difference-in-difference models, we estimated the effect of AMNOG on launch delay, the ranking order of launch delays, and the availability of pharmaceuticals. We then compared the results for Germany to Austria, Czechia, Italy, Portugal, and the UK. Across all six countries, launch delays decreased from the pre-AMNOG period (25.01 months) to the post-AMNOG period (14.34 months). However, the introduction of AMNOG consistently reduced the magnitude of the decrease in launch delay in Germany compared to the comparator countries (staggered DiD: + 4.31 months, *p* = 0.05). Our logit results indicate that the availability of pharmaceuticals in Germany increased as a result of AMNOG (staggered logit: + 5.78%, *p* = 0.009). We provide evidence on the trade-off between regulation and access. This can help policymakers make better-informed decisions to strike the right balance between cost savings achieved through HTA based regulation and access to pharmaceuticals.

## Introduction

In response to years of rapidly rising pharmaceutical costs, many countries have introduced health technology assessment (HTA) as a ‘fourth hurdle’ to the market entry of pharmaceuticals over the past two decades [1–3]. The aim of HTA is to evaluate the relative advantages of newly introduced pharmaceuticals over existing pharmaceuticals in terms of effectiveness and costs. Because information from HTA can help decision-makers curb costs through measures such as price negotiations [4], critics of this fourth hurdle have speculated that HTA may compromise access to pharmaceuticals and therefore negatively affect patient care [5, 6].

The timing of the launch of a new drug is an important factor in getting the drug to the patient as quickly as possible. New pharmaceuticals generally imply better management and treatment of many illnesses, and it is widely recognised that access to modern medical treatments, including new pharmaceuticals, has contributed immensely to improving patient outcomes [7]. Delays in the launch of new pharmaceuticals can result in some patients receiving inadequate care, leading to the loss of life years and lower quality of life [8, 9], especially if the difference between the new treatment and the current standard of care is large, or if the standard of care is contraindicated or cannot be tolerated by a given patient. Additionally, poorer access to new pharmaceuticals shifts the volume of treatments prescribed to older molecules with a potentially lower therapeutic value [10], in turn leading to higher expenditure on other forms of medical care [11, 12]. For patients, a delay in the launch of a new pharmaceutical can be costly if they must have it imported from another country and/or pay for it out of pocket. In addition, in cases where a delay is not due to a deliberate decision, it can also be a problem for the manufacturer because it shortens the time available to refinance development costs.

Despite the clear importance of delays in drug launches, their dynamics over time and possible cause-and-effect relationships with regulatory factors have received little research attention to date [13]. Moreover, there is little evidence on whether HTA itself which can be regarded as a form of strict regulation could lead to longer launch delays and poorer access to pharmaceuticals in the first place. Studies have shown that delays in the launch of new pharmaceuticals increased in the United States (US) following the introduction of the ‘third hurdle’ requirement to prove their safety, quality, and efficacy by the 1962 Amendments to the Food, Drug and Cosmetics Act [14, 15]. Additionally, and more generally, prior research suggests that countries that regulate prices more aggressively or in which the market size of a product is expected to be small have fewer launches and longer launch delays [16]. Empirical studies showing a causal relationship between HTA and access to pharmaceuticals are, however, still lacking.

To address these gaps in the literature, we used the introduction of Germany’s HTA system through the Pharmaceutical Market Restructuring Act (AMNOG) in 2011 [17] to analyse whether HTA-based regulation in Germany has a causal effect on access to pharmaceuticals. HTA has already been carried out before (e.g. DIMDI, IQWIG, etc.) in Germany, but not as a mandatory requirement for every new prescription pharmaceutical. By using the AMNOG as an example for HTA-based regulation we looked in particular at the length of the launch delays -as this is often used as a proxy for patient access in research [16, 18–23], changes in the ranking order of launch delays across countries, and the availability of new pharmaceuticals in each country before and after the introduction of the German HTA system. This system takes a two-stage approach in which evidence-based assessments of the medical benefits of a new pharmaceutical are undertaken using data from prior clinical trials and serve as the basis for price negotiations that start 6 months after a pharmaceutical has been launched; in the interim, the manufacturer may set the price for the pharmaceutical and this is reimbursed in full by statutory health insurance [24]. This being said, the AMNOG is primarily a form of price regulation and only affects reimbursement indirectly. In contrast to the National Institute for Health and Care Excellence (NICE) in England and Wales, the German HTA system does not consider quality-adjusted life years (QALYs)/cost-effectiveness in its final decision, and the average duration of the process in Germany is shorter (German HTA system: 6 months, NICE: 9–12 months) [25].

## Methods

### Data

The first international launch date (worldwide) and national launch dates of prescription pharmaceuticals in the following 30 European countries were extracted from the IQVIA (former IMS) MIDAS Sales Database: Austria, Belgium, Bosnia-Herzegovina, Bulgaria, Croatia, Czechia, Estonia, Finland, France, Germany, Greece, Hungary, Ireland, Italy, Latvia, Lithuania, Luxembourg, Netherlands, Norway, Poland, Portugal, Romania, Serbia, Slovakia, Slovenia, Spain, Sweden, Switzerland, Turkey, and the United Kingdom (UK). IQVIA collects these data from two major sources: wholesalers and hospital pharmacies. The IQVIA dataset has been used for related research before [26–28].

We restricted the sample to prescription pharmaceuticals that were launched between 2003 and 2017 to have a solid base covering both a pre-AMNOG period (2003–2009) and a post-AMNOG period (2011–2017). For national launch dates, our follow-up period was until the second calendar quarter of 2020. We excluded data from the year 2010 from our analyses because the announcement of the new HTA-based regulation system was published in that year, and companies may have attempted to launch products more quickly after the announcement to avoid these being subject to the new regulation starting in 2011.

To avoid confounding (a) by pharmaceuticals being launched very late in some countries after already having been taken from the market elsewhere, or by (b) peculiarities in how certain substances are defined as a (prescription) drug in some countries, two researchers independently reviewed all pharmaceuticals that were launched in fewer than five countries to verify the launch dates in the database. Furthermore, we excluded vaccines from the analyses because these are not regulated via the AMNOG.

We operationalized access to pharmaceuticals in three ways: (I) *Launch delay*, which we calculated as the length of time between the first international launch date of each pharmaceutical and its corresponding national launch date in a given country; (II) changes in the *ranking order of launch delays* across all 30 countries in our sample (with the country with the shortest launch delay being ranked first and the country with the longest launch delay being ranked thirtieth, etc.); (III) differences in the *availability of pharmaceuticals* before and after the introduction of HTA-based regulation in Germany; we calculated these by determining the number of pharmaceuticals launched internationally during the two intervals (i.e., 2003–2009 and 2011–2017) and calculating the percentage of these pharmaceuticals that were available in a given country during each interval. Furthermore, we differentiated between potential blockbusters (defined as bestselling products) and all other products. We have defined a pharmaceutical as a bestseller if it was among the top 50 blockbusters worldwide in the last two decades. Drugs that were defined as bestseller drugs (ranked by sales in Mio. USD) can be found in appendix (Table 4).

### Empirical model

To assess the impact of HTA on access to new pharmaceuticals in Germany, we calculated the values for our three measures of access for Germany for the pre-AMNOG and post-AMNOG period. We then compared these to those in five EU centralized and regulated countries that (a) had not had any major regulatory interventions related to HTA or reimbursement after 2011 and (b) had a similar pre-AMNOG trend from 2003 to 2009. These countries were Austria, Czechia, Italy, Portugal, and the UK. As of late 2021, Austria has used a reimbursement system consisting of three tiers subsequent to price setting since 2005 [29]. In Czechia, the State Institute for Drug Control has been responsible for pricing and reimbursement decisions since 2008 and manages reimbursement the same way [30]. In Italy, AIFA (Agenzia Italiana del Farmaco) has assumed the role of HTA agency and negotiated prices on behalf of the Italian NHS (National Health Service) in a decentralised system since 2003 [31]. In Portugal, INFARMED (Instituto Nacional da Farmácia e do Medicamento) and (since 2015) SINATS (Sistema Nacional de Avaliação de Tecnologias de Saúde) has conducted HTA of pharmaceuticals pertaining to their pricing and reimbursement; additionally, external reference pricing has been used since 2003 [32]. The UK introduced HTA procedures as part of the remit of the National Institute for Clinical Excellence (now the National Institute for Health and Care Excellence) in 1999 in England and Wales but has not made any major changes since then [33]. Most importantly, there has not been any major change to HTA-based regulation in Germany since the AMNOG legislation came into force in 2011 [34]. Also, there was no major intervention from 2009 on in the other countries that could have been effective from 2011 on.

To analyse changes in access to pharmaceuticals, i.e., (I) launch delay, (II) changes in the ranking order of launch delays, and (III) the availability of pharmaceuticals, we estimated two difference-in-difference (DiD) models (for (I) launch delay and the (II) ranking order of launches) and one logit model (for the (III) availability):

$${\mathrm{Access \,to \,pharmaceuticals}}_{i,t}={\beta }_{0}+ {\beta }_{1}* \mathrm{time}+ {\beta }_{2}* \mathrm{treated}+ {\beta }_{3}*\mathrm{ time}*\mathrm{ treated}+\varepsilon$$

Access to pharmaceuticals refers to our outcome of interest in country *i* at time *t* (launch delay, ranking order of launches, availability)*. *Time was coded as 0 if the first international launch of a pharmaceutical took place before the introduction of the German HTA system (2009 or earlier) and as 1 if the first international launch took place after its introduction (2011 or later). *Treated* was coded as 1 for Germany and 0 otherwise.

Parallel pre-trends were checked using (a) graphical inspection (for graphs see appendix Fig. 1, 2, 3, 4, 5) and (b) placebo regression (see appendix Table 5). We modelled a placebo regression as if the German HTA system had been introduced in 2008, making 2003–2007 the pre-AMNOG period and 2008–2009 the post-AMNOG period. We could not detect any significant coefficients in our interaction terms when using placebo regression, which supports the validity of our original model.

We further estimated a staggered DiD to aggregate the effects of introducing HTA-based regulation in Germany across countries:

$${\mathrm{Access \,to \,pharmaceuticals}}_{it}= {\mu }_{i}+ {\lambda }_{t}+ \delta {\mathrm{\,interaction \,term}}_{it}+ {\varepsilon }_{it}$$

with δ being the effect of the introduction of the HTA system in Germany. Furthermore, ${\mu }_{i}$ and ${\lambda }_{t}$ are country and time fixed effects. ${\varepsilon }_{it}$ is an unobserved error term.

For our subgroup analysis, we differentiated between bestseller drugs (ranked by sales in Mio. USD) and pharmaceuticals with lower sales. Furthermore, to differentiate between the short-, medium-, and long-term consequences of the introduction of the HTA system, we looked at three time intervals after the system came into effect: 2011–2013, 2014–2016, and 2017.

All analyses were performed using Stata SE 16. Analyses are computed based on the total number of non-missing cases. Our outcomes are thus conditioned on availability.

## Results

Our final sample included 492 different pharmaceuticals, 269 of which were launched in the pre-AMNOG period (2003–2009), and 223 of which were launched in the post-AMNOG period (2011–2017). In total, 37 (13.75%) of the pharmaceuticals we classified as bestsellers were launched in the pre-AMNOG period and 15 were launched in the post-AMNOG period (6.72%). Generally, we saw a trend towards a decrease in launch delay across Germany and all five comparator countries from the pre-AMNOG period to the post-AMNOG period. Among the six countries, the launch delay was 25.01 months on average in the pre-AMNOG period and 14.34 months on average in the post-AMNOG period.

In absolute figures, however, it was the countries with a large expected market size that launched earlier than countries with the small expected market size. Across our full sample of 30 countries, Germany's ranking worsened slightly from a rank 3.79 in the pre-AMNOG period to a rank 3.93 in the post-AMNOG period. For the five comparator countries, we found that Austria went from rank 8.63 in the pre-AMNOG period to rank 4.59 in the post-AMNOG period, Czechia from rank 16.94 to rank 16.75, Italy from rank 13.28 to rank 12.60, Portugal from rank 13.69 to rank 9.59, and the UK from rank 4.67 to rank 3.64.

With regard to the availability of new pharmaceuticals, in the pre-AMNOG period (2003–2009), we found that 221 (82.16%) of the 269 pharmaceuticals launched internationally were also launched in Germany. In the post-AMNOG period, this percentage increased, with 201 (90.13%) of the 223 products launched internationally also being launched in Germany. Among the five comparator countries, the availability of new pharmaceuticals increased from 78.07 to 83.86% in Austria, from 68.77 to 69.06% in Czechia, from 78.87 to 82.06% in Italy, and from 79.93 to 87.00% in the UK, but decreased from 75.84 to 70.40% in Portugal. Table 1 reports the descriptive characteristics of the specified variables for Germany and all five comparator countries.

**Table 1:** Dataset characteristics

	Austria	Czechia	Germany	Italy	Portugal	UK	Ø	SD
New molecules								
Before 2009	210	185	221	212	204	215	207.83	11.42
After 2011	187	154	201	183	157	194	179.33	17.78
Difference	23	31	20	29	47	21	28.50	9.20
Average launch delay (months)								
Before 2009	22.07	34.44	16.98	28.27	29.54	18.74	25.01	6.23
After 2011	9.91	22.15	9.84	18.51	16.04	9.59	14.34	4.89
Difference	12.16	12.29	7.14	9.76	13.5	9.15	10.67	2.18
Ranking order of launch delays								
Before 2009	8.63	16.94	3.79	13.28	13.69	4.67	10.17	4.85
After 2011	4.59	16.75	3.93	12.60	9.59	3.64	8.52	4.93
Difference	4.04	0.19	− 0.14	0.68	4.1	1.03	1.65	1.75
Availability of pharmaceuticals (%)								
Before 2009	78.07	68.77	82.16	78.87	75.84	79.93	77.27	4.25
After 2011	83.86	69.06	90.13	82.06	70.40	87.00	80.42	7.97
Difference	5.79	0.29	7.97	3.19	− 5.44	7.07	3.15	4.61

Our descriptive results were confirmed by our DiD models. In general, the launch delay in Germany and all five comparator countries decreased significantly from the pre-AMNOG period to the post-AMNOG period (-12.16 months for Austria, -12.29 months for Czechia, -7.14 months for Germany, -9.76 months for Italy, -13.50 months for Portugal, and -9.15 months for the UK). However, it appears that the introduction of AMNOG in Germany consistently reduced the magnitude of the decrease in launch delay compared to each of the five comparator countries (the coefficient for time*treat is always positive, see Table 2), although the reduction was not always statistically significant. More specifically, due to the introduction of AMNOG, the launch delay in Germany increased by + 5.02 months compared to Austria (*p* = 0.067), by + 5.15 months compared to Czechia (*p* = 0.081), by + 2.62 months compared to Italy (*p* = 0.316), by + 6.36 months compared to Portugal (*p* = 0.032) and by + 2.00 months compared to the UK (*p* = 0.437). The staggered DiD resulted in an estimate of + 4.31 months for Germany due to AMNOG. (*p* = 0.050).

**Table 2:** DiD Results: launch delay, ranking order of launch delays, and availability of new pharmaceuticals for Germany compared with Austria, Czechia, Italy, Portugal, and the UK

	Access to pharmaceuticals											
	DiD Germany vs. Austria		DiD Germany vs. Czechia		DiD Germany vs. Italy		DiD Germany vs. Portugal		DiD Germany vs. UK		Staggered DiD	
	Coef	SE	Coef	SE	Coef	SE	Coef	SE	Coef	SE	Coef	SE
(I) Launch delay												
_Cons	22.07	1.37***	34.44	1.51***	28.27	1.30***	29.54	1.47***	18.74	1.29***	26.35	0.65***
Time	− 12.16	1.96***	− 12.29	2.20***	− 9.76	1.87***	− 13.51	2.18***	− 9.14	1.83***	− 11.46	0.93***
Treated	− 5.08	1.92**	− 17.45	2.04***	− 11.28	1.82***	− 12.55	2.03***	− 1.75	1.81	− 9.36	1.54***
Time##treated	5.02	2.73*	5.15	2.94*	2.62	2.61	6.36	2.96**	2.00	2.57	4.31	2.19**
(II) Ranking order of launches												
_Cons	8.90	0.71***	16.81	1.01***	13.45	0.64***	13.00	0.76***	4.81	0.42***	11.40	0.67***
Time	− 4.33	1.14***	− 0.53	1.62	− 0.88	1.04	− 3.42	1.21***	− 1.24	0.67*	− 2.08	1.07**
Treated	− 5.09	1.00***	− 13.00	2.30***	− 9.63	0.91***	− 9.18	1.07***	− 1.00	0.59	− 7.58	1.64***
Time##treated	4.51	1.61***	0.71	2.18	1.06	1.47	3.61	1.72**	1.42	0.95	2.26	2.64
(III) Availability of pharmaceuticals												
_Cons	1.26	0.14***	0.78	0.13***	1.31	0.14***	1.14	0.14***	1.38	0.15***	1.16	0.06***
Time	0.37	0.23	0.01	0.19	0.20	0.22	− 0.27	0.20	0.51	0.25**	0.12	0.09
Treated	0.25	0.21	0.73	0.20***	0.21	0.21	0.38	0.21*	0.14	0.22	0.35	0.17**
Time##treated	0.30	0.36	0.67	0.33**	0.47	0.35	0.96	0.34***	0.16	0.37	0.55	0.29*
Margins	0.02	0.04	0.07	0.05	0.04	0.04	0.13	0.05***	0.01	0.04	0.05	0.03*

*p < 0.1

**p < 0.05

***p < 0.01

Across the larger sample of 30 countries, the ranking order of launch delays in Germany and the five comparator countries changed from the pre-AMNOG period to the post-AMNOG period. While Germany’s ranking order improved only slightly over time, that of the five comparator countries generally improved more substantially. Thus, our DiD models resulted in positive but, with the exception of Austria and Portugal, non-significant changes in the ranking order of launch delays. More specifically, due to the introduction of AMNOG, the ranking order of launch delay in Germany declined by + 4.51 ranks compared to Austria (*p* = 0.009), by + 0.71 ranks compared to Czechia (*p* = 0.758), by + 1.06 ranks compared to Italy (*p* = 0.474), by + 3.61 ranks compared to Portugal (*p* = 0.044), and by + 1.42 ranks compared to the UK (*p* = 0.146). The staggered DiD resulted in an estimate of an increase of 2.26 ranks for Germany due to AMNOG (*p* = 0.393).

With regard to the availability of new pharmaceuticals, our results indicate that AMNOG led to an increase in Germany compared to our comparator countries—i.e., by + 2.18% compared to Austria (*p* = 0.639), + 7.69% compared to Czechia (*p* = 0.138), + 4.72% compared to Italy (*p* = 0.317), + 13.41% compared to Portugal (*p* = 0.01), and + 0.90% compared to the UK (*p* = 0.841). The staggered logit resulted in an estimate of an increase of + 5.78% in the availability of new pharmaceuticals in Germany due to AMNOG (*p* = 0.099).

In our subgroup analyses distinguishing between bestsellers and pharmaceuticals with lower sales, the DiD results for bestsellers indicate that launch delay and the ranking order of launch delays increased to a smaller extent compared to pharmaceuticals with lower sales and that these were no longer significant. The effect sizes for the availability of new pharmaceuticals could not be computed for bestsellers as all bestseller were available in Germany and our 5 comparator countries (outcome does not vary).

The results of our subgroup analyses also indicate that the increase in launch delay seen in Germany was mostly stable or slightly larger when compared over the different post-AMNOG time intervals (see Table 3). For example, comparing Germany to Italy, we found an increase in launch delay by + 2.88 months (*p* = 0.398) in 2011–2013, by + 2.41 months (*p* = 0.466) in 2014–16 and + 4.18 months in 2017 (*p* = 0.444). The DiD results for changes in the ranking order of launch delays and logit results for the availability of new pharmaceuticals show a similar picture. The results from the staggered DiD/logit support the results from individual DiDs/logits.

**Table 3:** DiD Results for subgroup analysis (i.e. bestsellers and different time intervals after introduction of German HTA system)

	Subgroup analysis																								
	Post-AMNOG time intervals													Bestseller											
	DiD Germany vs. Austria		DiD Germany vs. Czechia		DiD Germany vs Italy		DiD Germany vs. Portugal			DiD Germany vs UK		Staggered DiD		DiD Germany vs. Austria		DiD Germany vs. Czechia		DiD Germany vs Italy		DiD Germany vs. Portugal		DiD Germany vs UK		Staggered DiD	
	Coef	SE	Coef	SE	Coef	SE	Coef	SE		Coef	SE	Coeff	SE	Coef	SE	Coef	SE	Coef	SE	Coef	SE	Coef	SE	Coeff	SE
(I) Launch delay																									
_Cons	22.07	1.33***	34.44	1.51***	28.27	1.30***	29.54	1.46***		18.74	1.28***	26.35	0.64***	85.77	10.49***	99.73	11.98***	92.78	9.93***	84.85	14.68***	83.83	10.27***	89.39	5.44***
Time														− 72.65	10.92***	− 78.22	12.40***	− 77.13	10.28***	− 66.69	15.19***	− 71.33	10.72***	− 72.94	5.64***
2011–2013	− 10.27	2.52***	− 8.25	2.75***	− 6.79	2.42***	− 9.37	2.86***		− 7.10	2.38***	− 8.27	1.21***												
2014–2016	− 13.17	2.40***	− 15.06	2.85***	− 11.24	2.39***	− 15.65	2.81***		− 10.43	2.36***	− 13.18	1.20***												
2017–2019	− 14.27	4.00***	− 19.74	5.30***	− 14.89	4.09***	− 19.85	4.73***		− 11.29	3.67***	− 16.26	2.00***												
Treated	− 5.08	1.92***	− 17.45	2.04***	− 11.28	1.81***	− 12.55	2.02***		− 1.75	1.80	− 9.36	1.53***	− 0.91	14.84	− 14.88	16.94**	− 7.93	14.04**	0.00	20.76	1.01	14.52	− 4.54	13.33
Time##treated														− 0.04	15.47	5.52	17.60	4.44	14.60	− 6.00	21.59	− 1.36	15.17	0.24	13.90
2011–2013	6.37	3.51*	4.35	3.78	2.88	3.41	5.46	3.90		3.19	3.38	4.36	2.82												
2014–2016	4.33	3.34	6.23	3.77*	2.41	3.31	6.82	3.78*		1.59	3.29	4.34	2.77												
2017–2019	3.56	5.52	9.02	6.58	4.18	5.45	9.14	6.19*		0.57	5.15	5.55	4.43												
(II) Ranking order of launches																									
_Cons	8.90	0.75***	16.81	1.04***	13.45	0.68***	13.00	0.80***		4.81	0.44***	11.40	0.68***	4.22	0.47***	9.81	0.75***	8.78	0.64***	6.71	0.68***	4.27	0.56***	6.90	0.36***
Time														− 0.28	0.71	− 1.46	1.20	− 2.85	0.94***	0.21	1.07	− 1.50	0.87*	− 1.29	0.56**
2011–2013	− 4.90	1.62	1.51	2.26	− 1.12	1.47	− 2.66	1.73		− 0.81	0.95	− 1.60	1.47												
2014–2016	− 4.24	1.62	− 2.48	2.26	− 1.12	1.47	− 3.66	1.73**		− 1.81	0.95*	− 2.66	1.47*												
2017–2019	− 2.90	2.60	− 0.81	3.62	0.54	2.37	− 5.00	2.77*		− 0.81	1.52	− 1.80	2.37												
Treated	− 5.09	1.06***	− 13.00	1.47***	− 9.63	0.96***	− 9.18	1.13***		− 1.00	0.62	− 7.58	1.67***	− 0.63	0.68	− 6.22	1.13***	− 5.20	0.93***	− 3.12	1.01***	− 0.68	0.80	− 3.31	0.95***
Time##treated														− 0.15	1.02	1.01	1.75	2.40	1.38*	− 0.65	1.55	1.06	1.22	0.85	1.42
2011–2013	5.42	2.29	− 1.00	3.19	1.63	2.09	3.18	2.44		1.33	1.34	2.11	3.62												
2014–2016	4.42	2.29	2.66	3.20	1.30	2.09	3.84	2.44		2.00	1.34	2.84	3.62												
2017–2019	2.09	3.68	0.00	5.12	-1.36	3.35	4.18	3.92		0.00	2.16	0.98	5.81												
(III) Availability of pharmaceuticals																									
_Cons	1.26	0.14***	0.78	0.13***	1.31	0.14***	1.14	0.14***		1.38	0.15***	1.16	0.06***												
Time																									
2011–2013	0.33	0.31	0.66	0.29**	0.65	0.35 *	-0.07	0.28		0.94	0.40**	0.45	0.14***												
2014–2016	0.41	0.30	− 0.18	0.24	0.09	0.29	-0.36	0.25		0.15	0.30	− 0.01	0.12												
2017–2019	0.37	0.51	− 0.85	0.38**	-0.41	0.42	-0.54	0.40		0.85	0.62	− 0.24	0.18												
Treated	0.25	0.21	0.73	0.20***	0.21	0.21	0.38	0.21*		0.14	0.22	0.35	0.17**												
Time##treated																									
2011–2013	0.21	0.48	− 0.11	0.47	− 0.10	0.51	0.62	0.46		− 0.39	0.54	0.09	0.39												
2014–2016	0.28	0.48	0.87	0.44**	0.59	0.46	1.05	0.44**		0.53	0.47	0.70	0.38*												
2017–2019	0.76	0.90	2.00	0.84**	1.56	0.85*	1.69	0.84**		0.29	0.97	1.39	0.77*												
Margins	0.01	0.02	0.07	0.02***	0.04	0.02*	0.08	0.02***		0.01	0.02	0.04	0.02**												

*p < 0.1

**p < 0.05

***p < 0.01

## Discussion

In this study, we analysed the impact of health technology assessment (HTA) on access to pharmaceuticals using the introduction of Germany’s HTA system through the Pharmaceutical Market Restructuring Act (AMNOG) in 2011 as an example. We used difference-in-difference models to look at three different outcomes measuring access in Germany and five comparator countries: (I) launch delay, (II) the ranking order of launch delays across Europe, and (III) the availability of new pharmaceuticals. Although we found that launch delay generally decreased from the pre- to the post-AMNOG period in all six countries, we found that the decrease was significantly smaller in Germany due to the introduction of AMNOG. We also observed (minor) changes in the ranking order of launch delays that were consistent with these results. Thus, our study provides important evidence that HTA-based regulation can negatively affect access to pharmaceuticals when this is measured in terms of a launch delay. When looking at the availability of new pharmaceuticals, however, the effect sizes for Germany increased compared to those in the five comparator countries.

Although this is, to the best of our knowledge, the first study to investigate the causal link between HTA-based regulation and access to pharmaceuticals, our results are in accordance with related literature. In the US, for example, the Kefauver-Harris amendment to the Food, Drug, and Cosmetic Act in 1962 was shown to be associated with longer launch delays in that market [14, 15]. In England and Wales, HTA was introduced with the creation of NICE in 1999, which considers comparative effectiveness and cost-effectiveness data when evaluating new drugs. Unfortunately, no research has been undertaken on the impact of HTAs conducted by NICE on launch delay so far, but there are studies showing that positive guidance issued by NICE does not necessarily eradicate inequalities in access [35]. In research on drugs to treat orphan diseases, it has been found that while more than a half of the centrally approved pharmaceuticals were on the market in the investigated countries, patients’ access to orphan medical products was restricted by different national reimbursement policies, especially in the UK, Italy and Spain [36].

Furthermore, using country-specific average prices as an indirect measure for regulation, it has been shown that stricter (price) regulation is correlated with increased launch delay [37]. Also, because a low price in one market can spill over to other markets through parallel trading and external reference pricing [38], it appears reasonable for manufacturers to prefer a longer launch delay in certain countries if negotiations after HTA in one country results in a lower price. In the case of AMNOG, these negotiations must be completed within 12 months after the product has been launched. Another study that investigated the effect of price regulation on delays in the launch of new drugs found that countries with lower expected prices and smaller expected market size have fewer launches (i.e., less availability) and longer launch delays [16]. However, it should be noted that the pharma world has significantly changed in the past two decades. Several processes were optimised globally (logistics, manufacturing, economies of scale, etc.) which impacted launch delay in general and is visible in the downward time trend. However, sensitivity analysis also confirm that the effect of HTA-based regulation was consistent when judged upon in different time intervals (i.e. in the first, second and last three years after AMNOG). Also, with stricter regulation, pharmaceutical manufacturers earn less over time, so they have had an incentive to push into the market faster.

The German HTA system is unique in that HTAs are undertaken while products are already on the market and being fully reimbursed by insurers. This helps ensure that patients have quick access to new pharmaceuticals and may also serve as an incentive for companies to innovate [39], while also including a mechanism to contain costs after 12 months. This being said, manufacturers must deal with a large amount of bureaucracy when submitting evidence for the benefit assessment. For example, a dossier consisting of four modules with a total of around 1000 pages per new drug has to be submitted to a body known as the Federal Joint Committee (G-BA), a process that takes anywhere from six to 12 months to prepare [40]. If manufacturers choose to start this process very early when EMA approval is still uncertain, they run the risk of wasting resources in the event that approval is not obtained. Indeed, this may be one of the reasons for the later launches we observed in our sample, although the AMNOG system itself guarantees immediate market access.

In Germany, a new drug can be accessed immediately at launch and is reimbursed for all indications by statutory health insurance funds. However, this is not the case in every country. In some countries, only parts of the indication are reimbursed, the drug is not reimbursed at all or is reimbursed years later. Thus, our measure of launch delay -based on launch dates and not reimbursement dates- has certain limits when it comes to evaluating patient access. However, we concentrate on changes in access and these restrictions did also apply to the Pre-AMNOG-period.

We do not consider any withdrawals from the market. The opportunity of ‘opting-out’ in Germany that allows the manufacturer to withdraw the pharmaceutical from the market might thus overestimate results for availability in favour of HTA-based regulation. This is because in our post-observation period 22 pharmaceuticals were withdrawn from the market in Germany [41] which is about 10.9% of all launched products. It could be that those drugs would have not been launched in the pre-AMNOG-period anyway and are responsible for the increase in availability from 82.2 to 90.1% from the pre- to the post-period. However, since our data does not allow us to observe withdrawals in any other market, this is highly speculative.

HTA involves important trade-offs for individual patients and society alike: On the one hand, lower prices come at the cost of later access to pharmaceuticals, potentially leading to an increase in the number of life-years lost. On the other hand, lower prices mean that a health care system will save money in the long run, potentially allowing for spending in other areas that could lead to a gain in life years or other beneficial outcomes for society. Also, prior research has shown that a positive HTA evaluation may be seen by physicians as a positive rating of a pharmaceutical’s quality from a trusted third-party source, thereby increasing the speed with which the pharmaceutical is diffused [42]. This faster diffusion could at least partly compensate for the effects of a delayed launch.

For the pharmaceutical industry, HTA comes with an important trade-off, as well: Manufacturers must either undergo the HTA process and potentially accept lower prices for a new pharmaceutical, or they must cope with a reduced market size for their product. In fact, since the introduction of the German HTA system, there have been more market withdrawals in Germany than ever before [43]. However, it is important to put this observation into perspective and consider that (a) a large share of these market withdrawals have involved drugs that had no added benefit compared to an appropriate comparator [44] and (b) in general, more pharmaceuticals were launched in the post-AMNOG period (269) than in the pre-AMNOG period (223) in Germany.

A widely accepted problem described in the related literature is that pricing and reimbursement policies are frequently assessed in terms of their financial consequences, including their ability to contain costs, but less frequently in terms of their effects on access to care [45]. There is an urgent need for policymakers to take this point into account when examining the trade-offs of new regulations pertaining to HTA. For example, future researchers may wish to investigate whether there are variations among different indication groups with respect to the launch delay caused by HTA-based regulation, for example in oncology [46, 47]. We were not able to investigate this with our data set because we could not filter pharmaceuticals by ATC codes or indication.

Our DiD results distinguishing between bestsellers and pharmaceuticals with fewer sales need to be interpreted with caution. Interestingly, the effect sizes across all three indicators decreased and/or were no longer statistically significant. It may therefore be the case that AMNOG does not have the same impact on all pharmaceuticals, but rather has a stronger effect on pharmaceuticals with lower sales (e.g. through the long bureaucratic process or because the bestsellers are meant to be on the market as fast as possible anyway).

### Limitations

Our analyses have several limitations. First, there may have been small regulatory changes in Germany and the five comparator countries that took place during the post-AMNOG period. Although we screened the literature very carefully for large changes in HTA and reimbursement regulations, we may have overlooked small changes, such as increases in co-payments, that could have affected our results. This being said, the general trend in all six countries has been in the same direction: towards stricter regulation. Although small regulatory changes will not have happened at the same time or have been of the same magnitude in each country, the robustness of our results when these were differentiated by time intervals can be interpreted as a sign that any bias from this source is minor.

Second, we cannot draw any conclusions about whether pharmaceuticals whose launch was delayed might serve an urgent need on the market—for example, whether the pharmaceuticals were first-in-class innovations or me-too drugs. However, our logit results suggest that the availability of bestseller pharmaceuticals increased in the post-AMNOG period, and this finding is worthy of further investigation.

Third, the ranking order of launch delays changed only slightly for Germany in absolute terms (i.e., from rank 3.79 pre-AMNOG to rank 3.93 post-AMNOG), whereas the five comparator countries showed much bigger changes (e.g., Portugal went from rank 13.69 pre-AMNOG to rank 9.59 post-AMNOG), which are, of course, driving our empirical results. Nevertheless, compared to a country like Germany that started at rank 3.78, it should be noted that there is much more scope for improvement in countries with an initially high number in the ranking and a comparatively longer launch delay. However, our results also hold if Germany is compared to the UK, which started at a similarly rank as Germany in terms of a launch delay. In addition, looking at the two countries with on average the earliest launch in Europe (The Netherlands and Sweden), that were not used as comparators due to lack of parallel pre-trends, we see that they reduced launch delay from 9.51 to 5.35 months (The Netherlands) and from 13.81 to 6.02 months (Sweden). Thus, the 9.84 months of average launch delay that were observed for Germany in the time period after 2011 does by no means represent the lowest launch delay possible. Moreover, Germany has never been the first country to launch (0% of our sample before AMNOG and in 0% of our sample after AMNOG). However, Germany has been the second country to launch in 17.19% of our sample before AMNOG, but in 0% of our sample after AMNOG.

Fourth, IQVIA’s methods for collecting data on launch dates differ slightly from country to country. For example, the launch dates for Estonia, Greece, and Luxembourg are calculated based on reports from the retail sector, whereas the launch dates for all other countries in our sample are calculated based on both the retail and hospital sectors. This does not present a problem for our analysis, however, given that Germany and the five comparator countries in our sample all used the latter method. However, when calculating the ranks of these six countries among the larger sample of 30 countries, there may have been a small bias.

Fifth, especially for pharmaceuticals whose market launch had not yet taken place in all countries, our approach to dealing with missing values might have had two consequences: For older pharmaceuticals that may be launched in a country rather late in the future, the launch delay may have been even longer than we measured. This could mean that the increase in launch delay in Germany due to AMNOG may be smaller than our results suggest. For very new pharmaceuticals, however, missing values during a time of generally decreasing launch delays will result in the opposite bias—i.e., a slightly inflated measure of launch delay due to AMNOG. Which of the two biases dominates in our analysis or whether they balance each other out is difficult to determine.

Finally, as mentioned previously in the methods section, two researchers independently reviewed all pharmaceuticals that were launched in fewer than five countries to verify the related launch dates in the database. Although carried out to the best of our abilities, a manual check is always subject to uncertainties. The fact that this review was conducted separately by the two reviewers should have reduced errors to a minimum, however.

## Conclusion

The findings of our study provide important evidence on the effects of HTA based regulation on access to pharmaceuticals, especially in terms of a launch delay, changes in the ranking order of launch delays among 30 European countries, and the market availability of new pharmaceuticals. In particular, it appears that the implementation of HTA systems like that introduced by the Pharmaceutical Market Restructuring Act (AMNOG) in 2011 may lead to longer launch delays and thus later patient access in Germany, while also having a significant impact on the availability of new pharmaceuticals -compared to other countries. Policymakers may wish to consider these findings when taking decisions about the trade-off between regulation and access to care. Future research should investigate whether the delay in the launch of new pharmaceuticals we detected in our study may undermine patient care, leading to a loss of life years and quality of life and, if so, to what extent. Additionally, further research should explore whether the current trade-off between lower prices for new pharmaceuticals and longer launch delays are in line with the preferences of the population.

## Figures and Tables

**Fig. 1:**
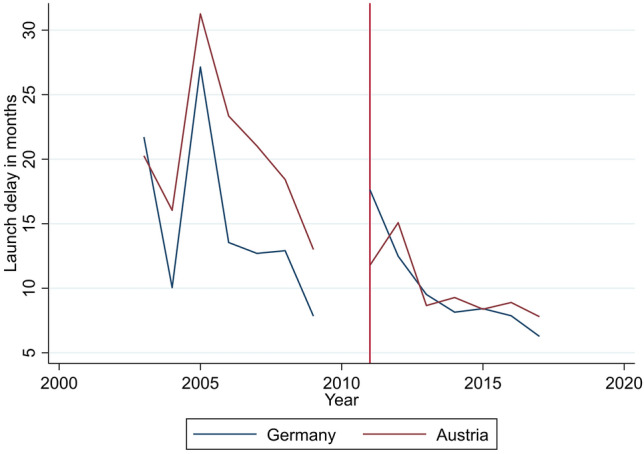
Germany vs. Austria

**Fig. 2:**
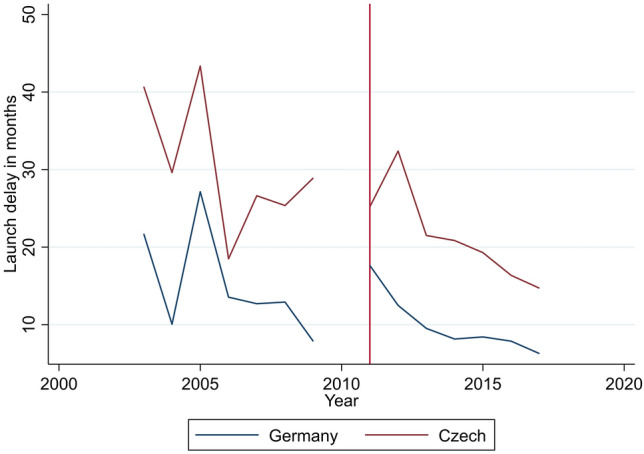
Germany vs. Czechia

**Fig. 3:**
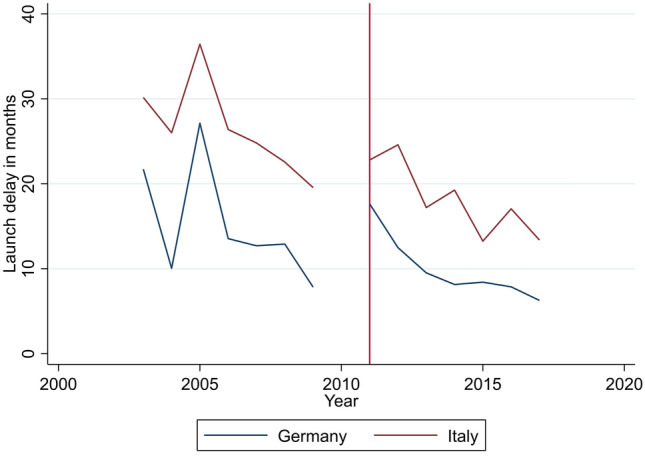
Germany vs. Italy

**Fig. 4:**
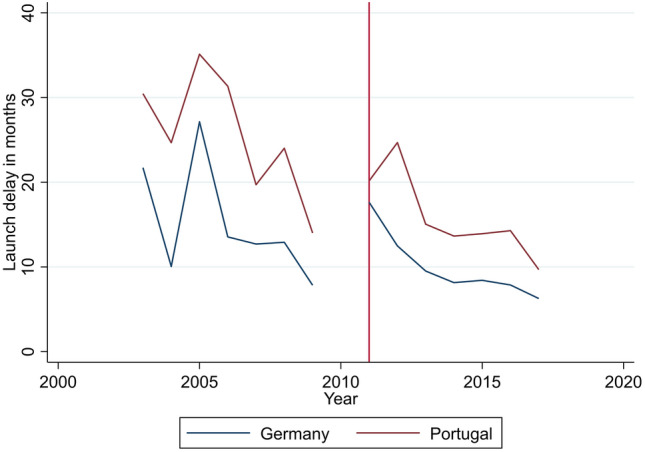
Germany vs. Portugal

**Fig. 5:**
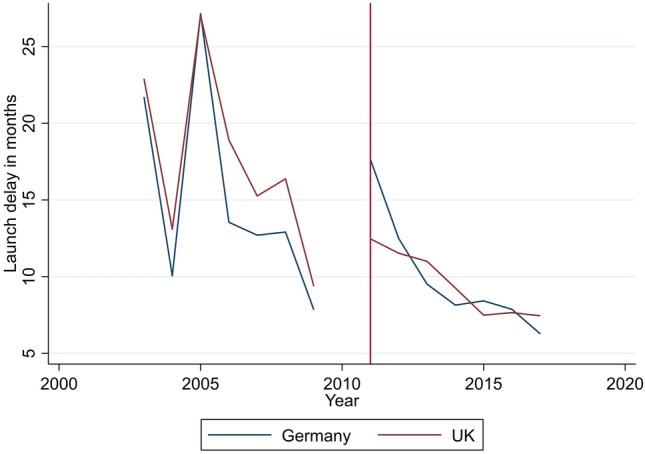
Germany vs. UK

**Fig. 6:**
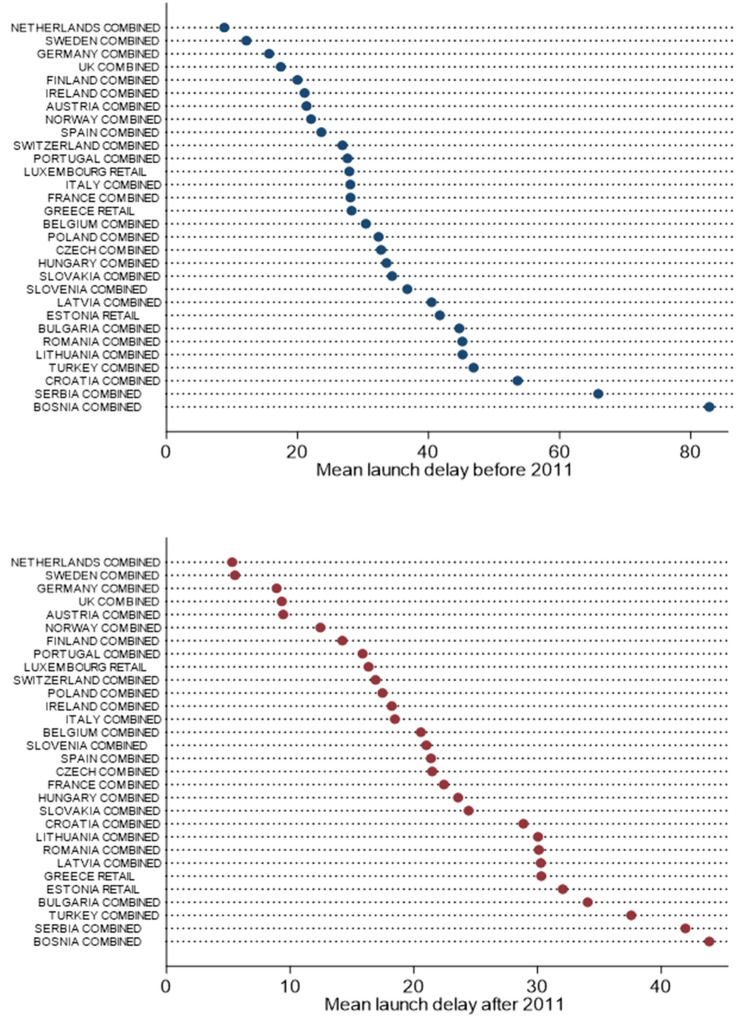
Launch order before and after 2011

## Data Availability

The data upon which our analysis (IQVIA, formerly IMS Health) is based are proprietary, and we used Stata SE 16 to conduct the analyses.

## Appendix

See Fig. 1, 2, 3, 4, 5, 6

See Tables 4, 5

**Table 4:** Bestseller

Pharmaceutical	Manufacturer	Total sales
Eliquis	Bristol-Myers Squibb/Pfizer2	$9.87 billion
Revlimid	Celgene	$9.69 billion
Keytruda	Merck	$7.17 billion
Enbrel	Amgen/Pfizer2	$7.13 billion
Herceptin	Roche	$6.84 billion
Eylea	Regeneron	$6.75 billion
Opdivo	Bristol-Myers Squibb	$6.74 billion
Avastin	Roche	$6.71 billion
Rituxin	Roche	$6.62 billion
Xarelto	Johnson & Johnson/Bayer2	$6.16 billion
Januvia/Janumet	Merck	$5.91 billion
Tivicay	GlaxoSmithKline	$5.88 billion
Remicade	Johnson & Johnson	$5.33 billion
Stelara	Johnson & Johnson	$5.16 billion
Genvoya	Gilead Sciences	$4.62 billion
Lyrica	Pfizer	$4.62 billion
Neulasta	Amgen	$4.48 billion
Tecfidera	Biogen	$4.27 billion
Lantus	Sanofi	$4.21 billion
Ibrance	Pfizer	$4.12 billion
Imbruvica	AbbVie	$3.59 billion
Juluca	GlaxoSmithKline	$3.52 billion
Mavyret	AbbVie	$3.44 billion
Gilenya	Novartis	$3.34 billion
Advair	GlaxoSmithKline	$3.22 billion
Trulicity	Eli Lilly	$3.20 billion
Humalog	Eli Lilly	$3.00 billion
Truvada	Gilead Sciences	$3.00 billion
Invega Sustenna	Johnson & Johnson	$2.93 billion
Cosentyx	Novartis	$2.84 billion
Perjeta	Roche	$2.72 billion
Orencia	Bristol-Myers Squibb	$2.71 billion
Symbicort	AstraZeneca	$2.56 billion
Avonex/Plegrity	Biogen	$2.36 billion
Ocrevus	Roche	$2.31 billion
Prolia	Amgen	$2.29 billion
Triumeq	GlaxoSmithKline	$2.18 billion
Alimta	Eli Lilly	$2.13 billion
Actemra	Roche	$2.12 billion
Simponi	Johnson & Johnson	$2.08 billion
Pomalyst	Celgene	$2.04 billion
Darzalex	Johnson & Johnson	$2.03 billion
Sprycel	Bristol-Myers Squibb	$2.00 billion
Humira	AbbVie	$19.9 billion
Epclusa	Gilead Sciences	$1.97 billion
Prezista	Johnson & Johnson	$1.96 billion
Aubagio	Sanofi	$1.94 billion
Aranesp	Amgen	$1.88 billion

**Table 5:** Placebo regression

	DiD		DiD		Access to pharmaceuticals DiD		DiD		DiD		Staggered DiD	
	Germany vs. Austria		Germany vs. Czech		Germany vs. Italy		Germany vs. Portugal		Germany vs. UK			
	Coef.	SE	Coef.	SE	Coef.	SE	Coef.	SE	Coef.	SE	Coef.	SE
(I) Launch delay												
_Cons	23.76	1.96***	36.19	2.07***	30.14	1.79***	31.69	1.96***	20.27	1.81***	28.17	0.88***
Time	− 8.39	4.37*	− 8.74	4.62*	− 9.35	4.02**	− 12.68	4.77**	− 7.84	4.10*	− 9.39	2.02***
Treated	− 5.09	2.72*	− 17.52	2.79***	− 11.47	2.50***	− 13.02	2.73 ***	− 1.60	2.54	− 9.49	2.10***
Time##treated	− 0.27	6.14	0.06	6.28	0.67	5.63	4.00	6.44	− 0.82	5.76	0.71	4.77
(II) Ranking order of launches												
_Cons	8.50	0.77***	17.37	1.44***	14.62	0.80***	14.62	0.83***	5.00	0.57***	12.02	0.77***
Time	− 2.00	1.73	− 0.87	3.22	− 4.62	1.79**	− 5.62	1.87**	− 0.50	1.29	− 2.63	1.00***
Treated	− 4.12	1.09**	− 13.00	2.04***	− 10.25	1.13***	− 10.25	1.18***	− 0.62	0.81	− 7.65	1.90***
Time##treated	0.12	2.45	− 1.00	4.56	2.75	2.53	3.75	2.65	− 1.37	1.83	1.76	2.46
(III) Availability of pharmaceuticals												
_Cons	1.32	0.17***	0.81	0.15***	1.36	0.17***	1.39	0.17***	1.52	0.18***	1.26	0.07***
Time	0.13	0.40	0.06	0.35	0.10	0.41	− 0.69	0.35**	− 0.05	0.41	− 0.10	0.17
Treated	0.38	0.26	0.89	0.25***	0.34	0.26	0.31	0.26	0.18	0.27	0.44	0.21**
Time##treated	− 0.23	0.59	− 0.17	0.56	− 0.20	0.59	0.59	0.56	− 0.04	0.60	0.00	0.46
Margins	− 0.03	0.08	− 0.02	0.09	− 0.03	0.08	0.12	0.09	0.00	0.08	0.00	0.06

**p* < 0.1

***p* < 0.05

****p* < 0.01
